# Numerical study on the hydrodynamic performance and internal flow of an axial-flow pump with various inlet flow angles

**DOI:** 10.1038/s41598-023-49671-4

**Published:** 2023-12-15

**Authors:** Yong-Jin Son, Yong-In Kim, Hyeon-Mo Yang, Kyoung-Yong Lee, Joon Yong Yoon, Young-Seok Choi

**Affiliations:** 1 Carbon Neutral Technology R&D Department, Research Institute of Clean Manufacturing System, Korea Institute of Industrial Technology, 89 Yangdaegiro-gil, Ipjang-Myeon, Seobuk-gu, Cheonan-si, 31056 Chungcheongnam-do Republic of Korea; 2 Department of Mechanical Engineering, Hanyang University, Seoul, 133-791 Republic of Korea; 3 Mechanical Engineering, Korea University of Technology and Education, 1600, Chungjeol-ro, Byeongcheon-Myeon, Dongnam-gu, Cheonan-si, Chungcheongnam-do Republic of Korea; 4 Industrial Technology (Green Processes and Energy System Engineering), University of Science and Technology, 217, Gajeong-ro, Yuseong-gu, Daejeon, 34113 Republic of Korea; 5 Aerospace Technology Research Institute-3rd Directorate Team 1, Agency for Defense Development, Yuseong, P.O. Box 35, Daejeon, 34186 Republic of Korea

**Keywords:** Mechanical engineering, Fluid dynamics

## Abstract

In this study, numerical simulation was employed to predict the performance and internal flow characteristics of the inlet of an axial-flow pump by assigning an absolute flow angle to the inlet guide vane (IGV) trailing-edge flow. Further, the finite volume method based on the three-dimensional Reynolds-averaged Navier–Stokes equations was employed to discretize the governing equations. The shear stress transport model was used as the turbulence model, and an appropriate number of nodes were selected for the hexahedral grid system through a grid-dependency test. The performance curve and changes in the internal flow field were investigated based on the variation in the flow angle at the inlet of the axial-flow pump. These results can be used to establish an efficient operational plan by adjusting the IGV angle of IGV when installing a variable IGV for an axial-flow pump.

## Introduction

Pumps, which are turbomachinery devices, utilize mechanical energy to increase the kinetic energy of fluids. Among them, axial-flow pumps are characterized by their generation of relatively low heads compared with those of centrifugal or mixed flow pumps; they are well suited for large-scale fluid transportation. Thus, they are primarily employed in water supply and drainage applications, as well as wastewater treatment plants. Typically, axial-flow pumps comprise components, such as an inlet guide vane (IGV), a bell-mouth-shaped suction casing, a propeller-blade-shaped impeller, and an outlet guide vane (GV) for static pressure recovery. The impeller, which is a key component, significantly influences the hydrodynamic performances of axial-flow pumps. Therefore, researchers have attempted to enhance the efficiencies and performances of pumps by optimizing the impeller design^1,2^. Compared with centrifugal pumps, axial-flow pumps induce an unstable operating region via the surge phenomenon, which arises from the variation in the slope of the total head (*H*) curve in the low-flow range. Numerous researchers have attempted to improve the performances of axial-flow pumps by ensuring stable operation. For example, Choi et al.^3^ researched the optimal variable-operating conditions for the efficient operation of axial-flow pumps exhibiting unstable-performance characteristics. Zierke et al.^4^ performed computational fluids dynamics (CFD) calculations on the three-dimensional (3D) turbulent flow of axial-flow pumps and compared the results with the experimental data. Thereafter, they constructed a database based on their analysis of the internal flow phenomena of the pump, thereby proposing smooth-operation methods for axial-flow pumps. Li et al.^5^ analyzed the internal flow patterns of axial-flow pumps to investigate their impact near the impeller on the hydrodynamic and energy-conversion performances of the pumps during operation. Based on the results, they further researched operational approaches. In response to load fluctuations, Draghici et al.^6^ proposed an optimal operational method by adjusting the rotation speed of the impeller within the pump.

Generally, it is crucial to avoid operating within the low-flow range because of the surge phenomena, which cause unstable hydrodynamic performances. Furthermore, efficient axial-flow pump operation can be achieved by installing IGV on the pump to increase its efficiency and reduce power consumption. IGV changes the angle of the incoming flow within the pump, thereby influencing the incidence angle. Consequently, IGV ensures control that can alter the performance characteristics of the pump, allowing it to adapt to changes in demand. To investigate this, Kaya et al.^7^ experimentally analyzed the impact of installing IGV on the performance characteristics of axial-flow pumps. They demonstrated how IGV installation improved the pump performance. Furthermore, they analyzed the variations in the internal flow patterns, as well as performance characteristics, of the pump based on the installation position of IGV. Additionally, they investigated the impacts of IGV installation, angle, and other factors on the performance curves and the shift of the best efficiency point^8–11^. Previous studies revealed that the installation of IGVs in axial-flow pumps changed the performance and efficiency characteristics of the pump. Qian et al.^12,13^ performed a comparative analysis of the variations in the internal flow, head, and efficiency of pumps based on the changes in the angle of the installed IGVs. Based on this analysis, they further investigated the implementation of IGVs on pump turbines in hydropower plants to analyze efficient operational strategies for pump turbines. Notably, the installation of a variable-geometry IGV in the internal section of an axial-flow pump benefits its performance and efficiency. Tang et al.^14^ analyzed the internal flow characteristics of an axial-flow pump by installing a variable-geometry IGV therein. They observed that the installation enhanced the efficiency (by ~ 10%) compared to the conventional setup. Numerous extant studies revealed that the IGV installation, which changes the absolute flow angle, influences the incidence angle^15,16^. Consequently, IGV was identified as a determining factor of pump performance; it is inferred that the installation of a variable-geometry IGV can influence the performance characteristics of a pump^17,18^. Qian et al.^19^ experimentally analyzed the impact of angle variations in a variable-geometry IGV on the performance characteristics of an axial-flow pump under off-design conditions, confirming that the optimization of the angle adjustment resulted in a maximum improvement of the pump efficiency (by 2.16%) and an expanded stable operating range. Xu et al.^20^ analyzed the variation in the energy performances, as well as the change in the stable operating range in low- and high-flow regions by numerically and experimentally altering the angle of a variable-geometry IGV that was installed in the pump. Numerous studies have considered the impact of installing variable-geometry IGVs on the performance characteristics of pumps.

Feng et al.^21^ experimentally analyzed the performance characteristics of axial-flow pumps equipped with variable-geometry IGVs, focusing on the effects of tip clearance variations in the IGVs. Kim et al.^22^ analyzed the effects of the thickness variations of axial-flow pump IGVs on the internal flow characteristics and performance parameters of the pimp. Liu et al.^23^ analyzed the internal flow distribution when adjusting the angle and axial distance of IGVs inside the pump and proposed the optimum installation angle afterward. Tan et al.^24,25^ investigated the impact of controlling pre-whirl with variable IGVs installed in pumps on the performance characteristics of the internal cavitation and unsteady flows of such pumps. There are ongoing active studies on the impacts of variable-geometry IGVs on the performance and efficiency characteristics of pumps^26,27^. As aforementioned, the extant studies mainly focused on the pump features after the installation of IGVs. However, the need for repetitive design modifications and production processes to improve pump performance and efficiency through IGVs may cause economic and time losses. Moreover, only a few studies have predicted the general impact of installed IGVs on the internal performance characteristics of pumps and presented valuable data for designing IGVs.

In this study, the swirling-flow-generated downstream of IGV to be installed at the inlet for the axial pump was numerically simulated. The shape of the inlet was simplified to minimize the effect on the flow angle flowing into the impeller during numerical analysis. The inlet flow angle corresponding to the design variable given an absolute flow angle of 0°, 5°, 15°, 25°, 35°, and 45° in the impeller rotation direction and the reverse direction of rotation, and the flow field characteristics were investigated using the 3D steady RANS equation. Thus, the surge phenomenon in the low-flow region during pump operation was identified, and this enabled the formulation of anticipated operating scenarios owing to fluctuations in the inflow rate, as well as the prevention of losses due to the design and production processes. It is still challenging to find systematic studies that predicted the effect of variable-geometry IGVs during the pump-design process and explored efficient operational strategies for energy conservation. Therefore, in this study, we analyzed the efficiency and flow field of axial-flow pumps equipped with variable-geometry IGVs using numerical simulations to predict the impacts of IGVs. Thereafter, we explored effective pump operation strategies based on our findings.

## Numerical analysis method

### Axial pump

**Figure 1:**
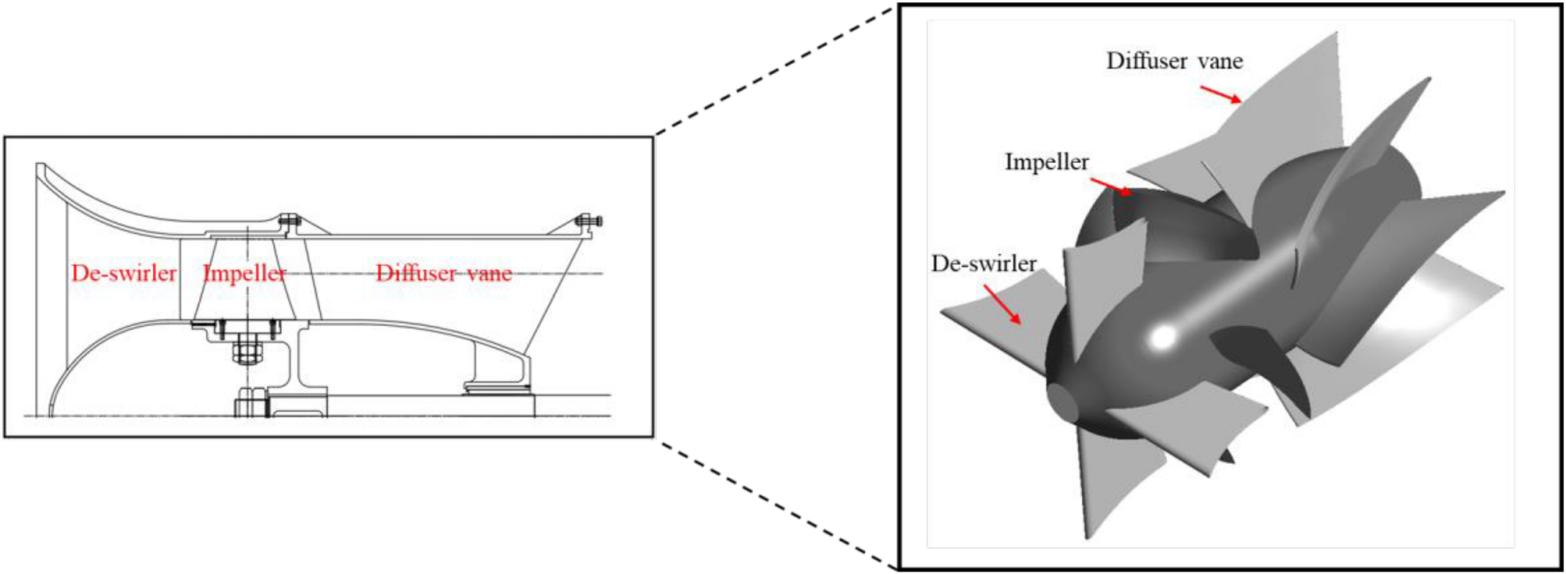
Meridional plane and 3D modeling of the axial-flow pump.

The axial-flow pump model, which is the subject of this study, comprises a de-swirler, an impeller, and diffuser vanes. The de-swirler supports the inlet casing and prevents the ingress of impurities into the impeller. The impeller boosts the pressure of the fluid entering through the inlet, whereas the diffuser vanes facilitate the recovery of the static pressure of the flow. The meridional, 3D model geometry of our subject is illustrated in Fig. 1. The detailed design specifications of the axial-flow pump are presented in Table 1, and the specific speed (${N}_{s}$) is calculated using Eq. (1). The flow rate, total head, and shaft power are non-dimensional based on Eqs. (2), (3), and (4) respectively.

**Table 1:** Design specification of the axial-flow pump.

Description	Value
Specific speed ($${N}_{s})$$	2.94
Flow coefficient ($\Phi )$	0.249
Head coefficient ($\Psi )$	0.239
Shaft power coefficient ($\lambda )$	0.079
Rotational speed (rpm)	2560
Impeller diameter (mm)	185
Number of de-swirlers	4
Number of impeller blades	4
Number of diffuser vanes	7

1 $${N}_{s}= \frac{\omega \sqrt{Q}}{{(gH)}^{3/4}}$$

2 $$\Phi = \frac{{c}_{m2}}{{u}_{2}}$$

3 $$\Psi = \frac{2gH}{{u}_{2}^{2}}$$

4 $$\lambda = \frac{L}{\frac{1}{2}\rho {A}_{2}{u}_{2}^{3}}$$

*Q *(m^3^/s), *H *(m), and *L *(W) represent the flow rate, total head, and shaft power, respectively. Further, $\omega , g, {C}_{m2}, {u}_{2}, \rho$, and *A* is represent the angular velocity, gravitational acceleration, meridional component of the absolute velocity at the impeller outlet, impeller outlet rotational velocity, density, and area, with units rad/s, m/s^2^, m/s, m/s, kg/m^3^, and m^2^, respectively.

### Inlet flow angle

**Figure 2:**
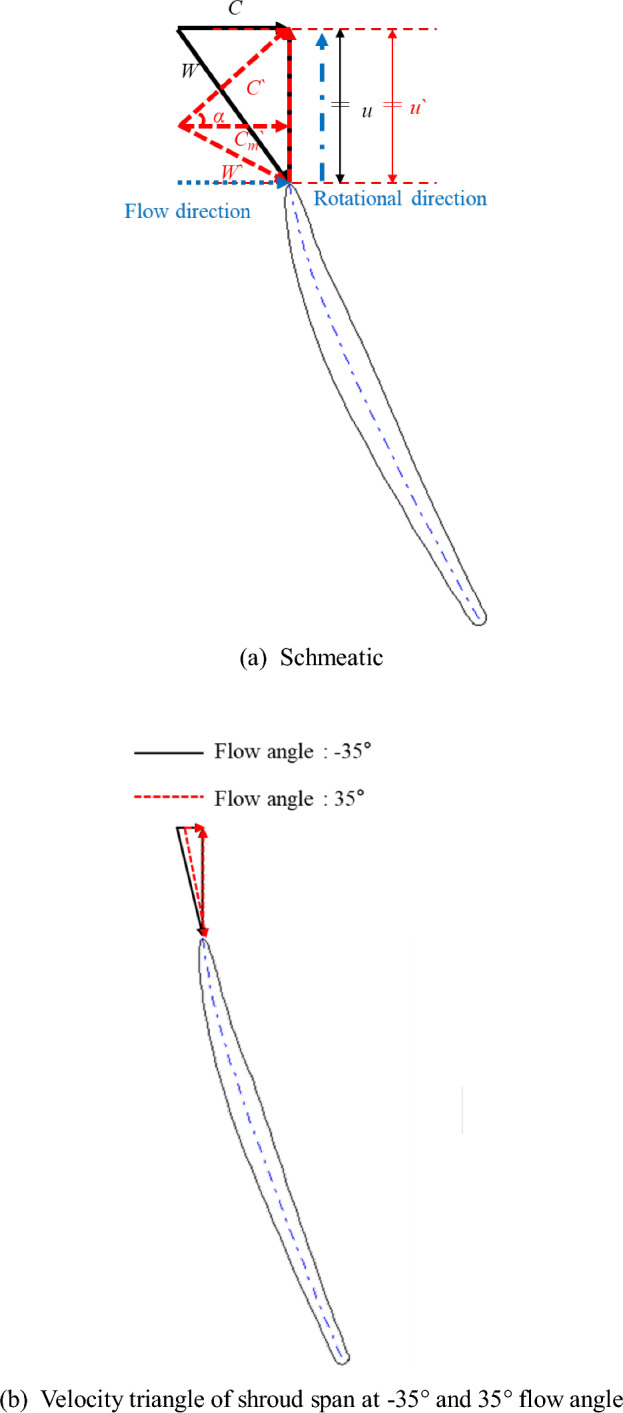
Velocity triangle at the impeller inlet (schematic).

The variable IGV, a component of an axial pump, changes the definite flow angle of the incoming fluid from the inlet, thereby influencing the incidence angle of the fluid entering the impeller and causing variations in the pump performance characteristics. Therefore, we simulated the variable IGV, which is installed at the inlet of an axial pump in the design process, by assigning a definite flow angle to the fluid at the pump inlet before installing IGV. The analysis was focused on investigating the impact of the inlet flow angle on the internal performance and flow characteristics of the pump. Figure 2a presents the schematic diagram of the velocity triangles in the inlet region of the impeller. Figure 2b shows the velocity triangle of the shroud span at the point of the best efficiency point impeller leading edge with inlet absolute flow angles of − 35° and 35°. For each vector, *c*, *u*, and *w* represent the absolute, tangential, and relative velocities, respectively. The solid black lines represent the operating condition under which IGV is not applied under the same flow rate and rotational velocity, whereas the red dashed lines represent the operating condition where IGV is applied. We assigned flow angles of 0°, 5°, 15°, 25°, 35°, and 45° with respect to the impeller rotation direction ( +), and we also assigned the same flow angles in the reverse direction of rotation ( −). The flow angle was indicated as the absolute flow angle (*α*) with respect to the axial direction, and it was applied to the inlet region of the investigated numerical analysis domain.

As shown in Fig. 2a, when the inlet absolute angle (α) changes under the same mass flow rate and rotational velocity conditions, the incidence angle changes. This change in the incidence angle affects the pump performance characteristics, and the flow rate point corresponding to the best efficiency point changes according to the change in the inlet flow angle. As can be seen in Fig. 2b, when the absolute flow angle applied to the inlet is changed, the total head and efficiency change due to the change in the flow rate point and incidence angle corresponding to the best efficiency point, Through this, performance characteristics change when the absolute flow angle applied to the inlet changes.

### Numerical analysis model and the boundary conditions

**Figure 3:**
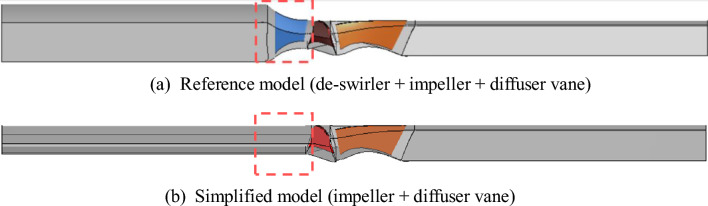
Numerical analysis domain.

The numerical analysis domain of this study was divided into the reference and simplified models. Figure 3 shows that the reference model comprised a bell-mouth-shaped inlet suction, a volute casing, an impeller, a diffuser vane, and an outlet section that were arranged from left to right in the flow direction. The rotating part could only be applied to the impeller region. In the future, the IGV may be installed at the de-swirler position. Moreover, as the absolute flow angle, which was assigned at the inlet during numerical analysis, can be influenced by the de-swirler, thereby affecting the flow angle of the fluid entering the impeller, the numerical analysis domain was simplified by excluding the geometry corresponding to the de-swirler. The simplified model extended the flow passage of the bell-mouth-shaped inlet section to the same diameter as that of the impeller inlet. The overall axial length of the numerical analysis domain was maintained the same as the reference model. The inlet and outlet sections were extended to a length that corresponded to quadruple the diameter to ensure numerical convergence in the numerical analysis.

**Figure 4:**
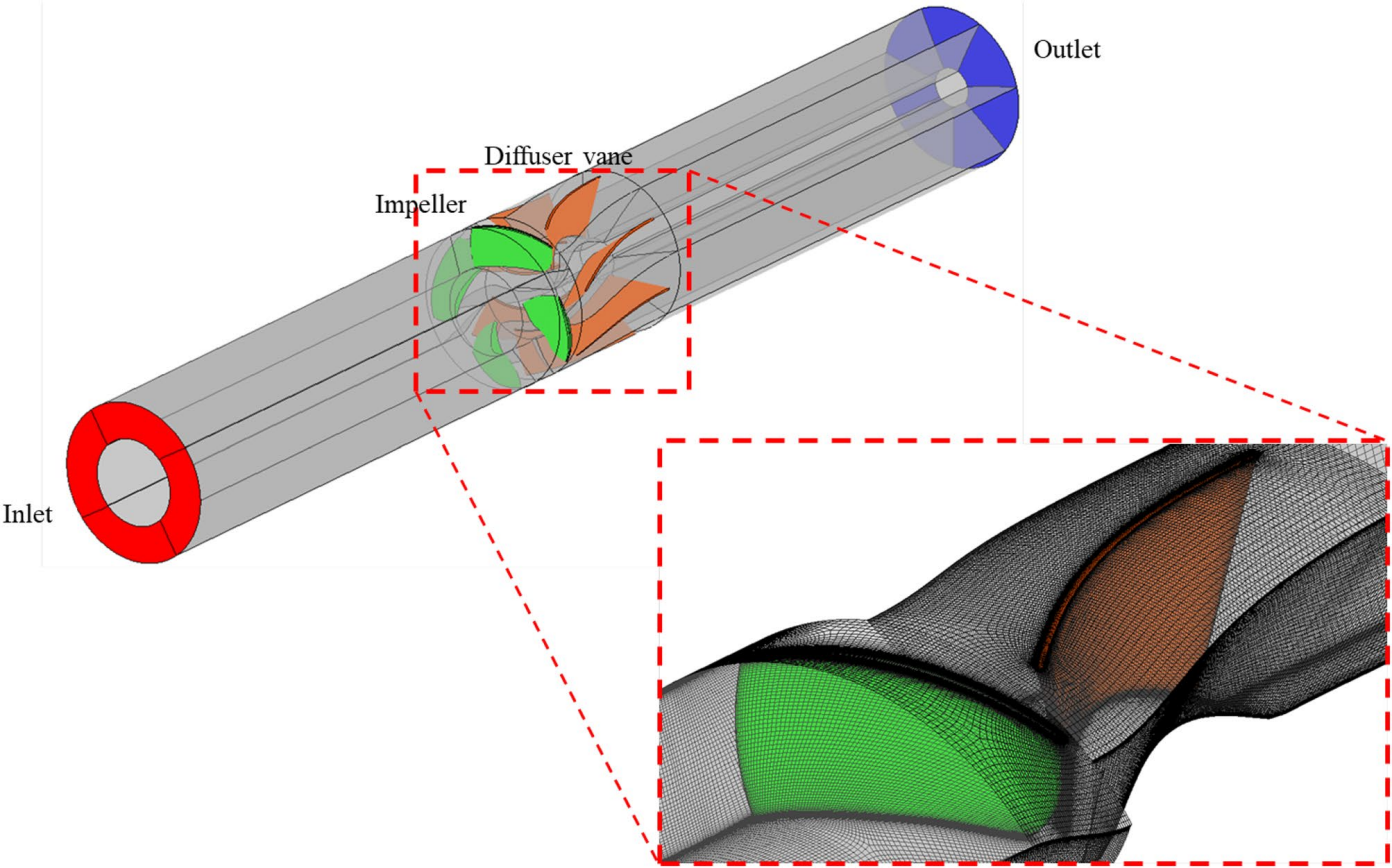
Geometry and grid system.

**Figure 5:**
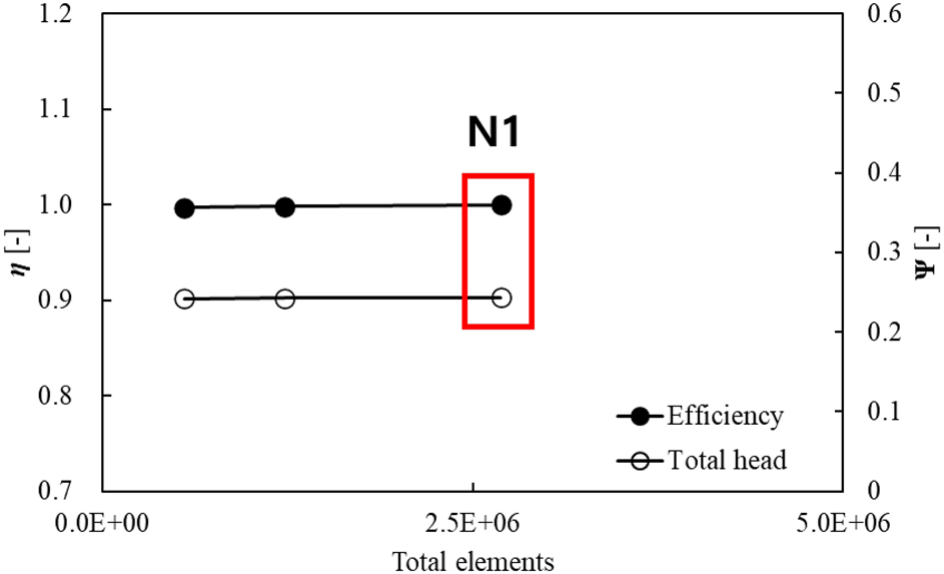
GCI results of the grid system of the base model.

Figure 4 shows the grid system in the numerical analysis; it comprised a structured grid of hexahedral elements. The grid system was validated by the grid convergence index (GCI) method, as proposed in a previous study^28^. For the quality of the grid, the y+ near the wall was kept below 2, and the average y+ of the blade surface was kept below 10. Grid verification was performed through the GCI method at the same y+ coefficient. Figure 5 shows the results of grid validation for the reference model (Fig. 3a); detailed information is presented in Table 2. We compared the computational errors in the efficiency and *H* across three observed grids at the design flow point. In this case, the efficiency was normalized using the computational values obtained from the grid system corresponding to N1. GCI of the optimized grid system (N1) comprising ~$2.7 \times {10}^{6}$ cells was ~ 0.0043, satisfying the previously proposed convergence criterion^28^. Therefore, we applied the grid system corresponding to N1.

**Table 2:** Calculation of the discretization error of the base model.

	$\mathrm{\varnothing }={\text{efficiency}}$	$H=\mathrm{total head}$
*N*_1_, *N*_2_, *N*_3_	2.7 × 10^6^, 1.2 × 10^6^, 5.5 × 10^5^	
*r*_21_	1.30	
*r*_32_	1.30	
*Ø*_1_	1	1
*Ø*_2_	0.9982	0.9993
*Ø*_2_	0.9969	0.9991
GCI_fine_^21^	0.0043	0.0028

Our numerical analysis was performed with the commercial CFD analysis software, ANSYS CFX-19.2^29^. To analyze the turbulence flow, the 3D steady Reynolds-averaged Navier–Stokes (RANS) equations were employed to investigate the flow field characteristics and the discretization was based on the finite volume method. The turbulence model incorporated the shear stress transport model^30,31^ to accurately analyze the flow separation phenomenon. The wall surface was subjected to no-slip and automatic wall-function conditions. For the uniform distribution of the absolute flow angle at the pump inlet, a mass flow condition was assigned to the inlet and atmospheric pressure condition was assigned to the outlet. Periodic conditions were applied to the rotational direction in the single-passage region to reduce computational time. The frozen rotor technique was employed between the stators, and the stage rotor technique was applied between the rotors and stators. The employed operating fluid was isothermal water, and the numerical analysis was performed using a 32-core dual-processor Xeon (2.8 GHz) central processing unit. The computational time for a single analysis was about 5 h.

### Evaluation of the computational model

**Figure 6:**
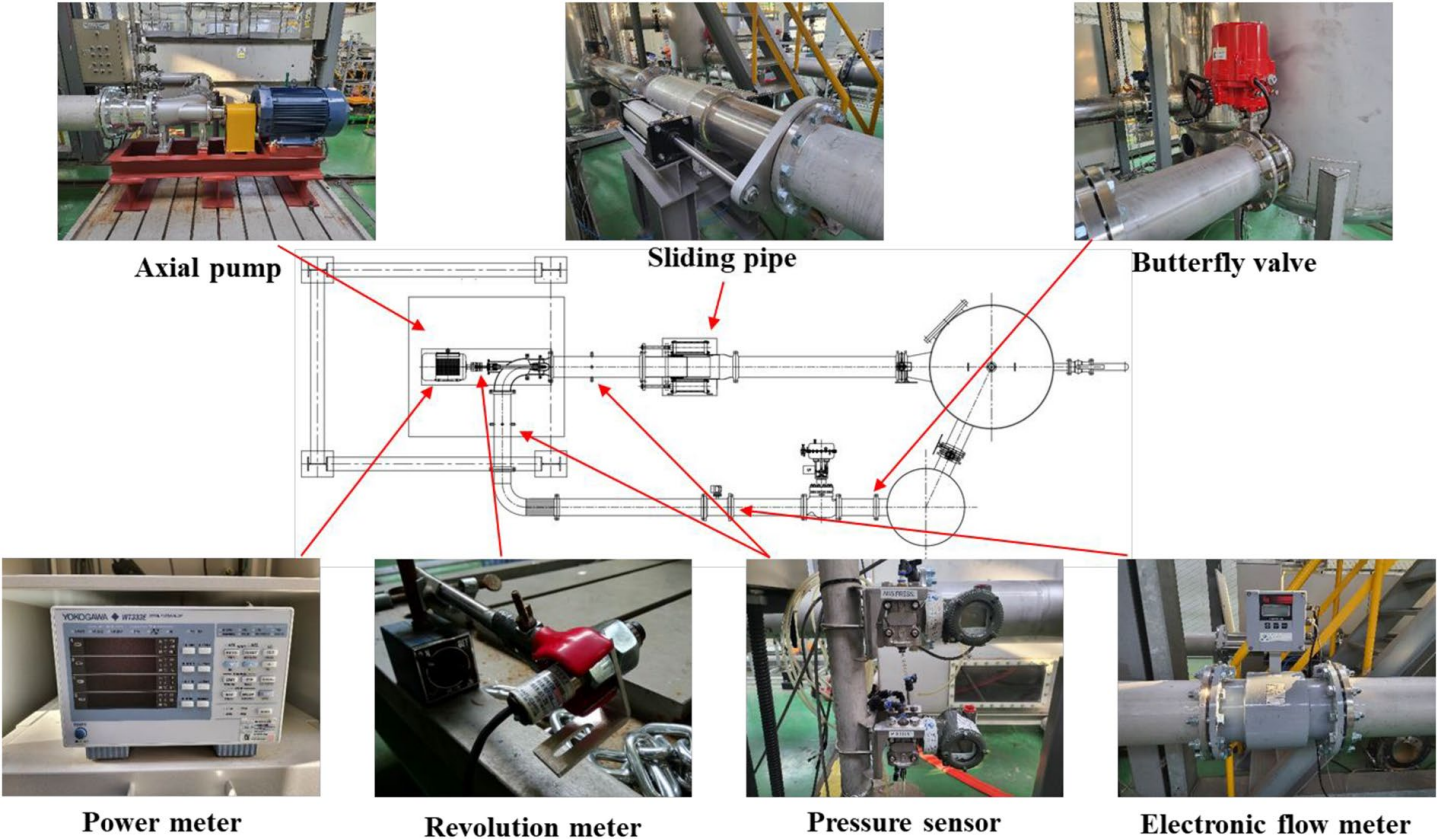
The schematic diagram for axial pump test facility and measurement device.

**Figure 7:**
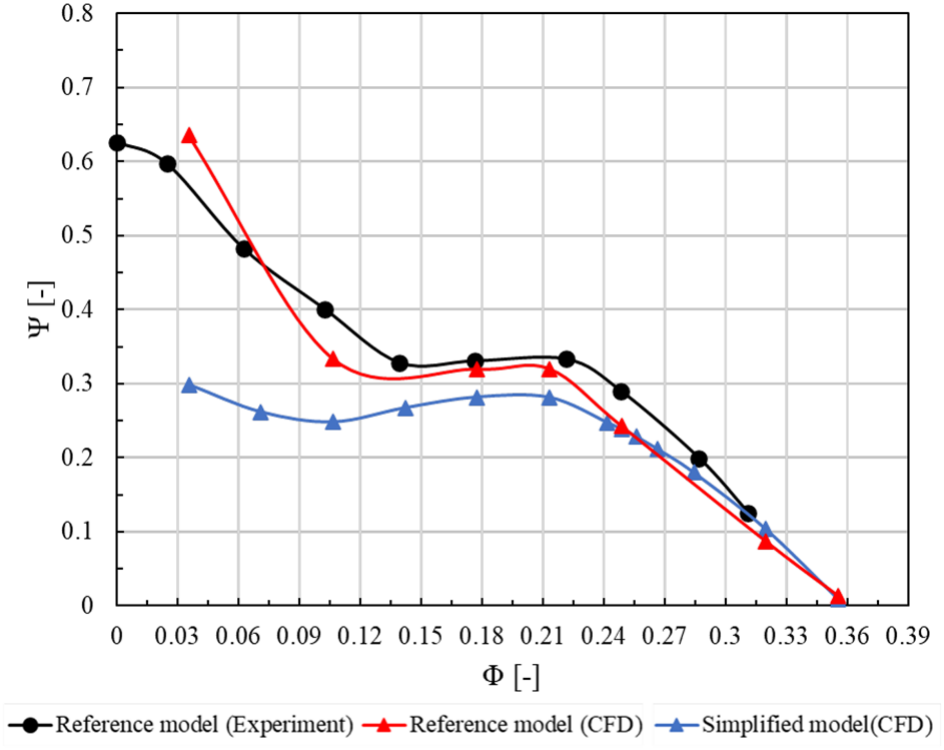
Comparison of the simulation and experimental results.

The experiment of the axial pump used in this study was carried out by the Korea Institute of Industrial Technology (KITECH), Korea. Figure 6 shows the schematic diagram and measurement equipment of the performance test equipment for evaluating the performance of the axial pump. The experiment was carried out on a closed loop test system with impellers installed. The test system includes an axial pump, a butterfly valve, a power meter, a revolution meter, a pressure sensor and an electronic flow meter. Equipment that meets the requirements of KS B6301 standard was used for the test range and precision of instrumentation items and instrumentation. Figure 7 shows the performance curve for the results of the numerical analysis of the reference and simplified models, as well as the experimentally obtained results of the performance test for the reference model. Overall, the experimental, and numerical analysis results of the reference model demonstrated consistent similarity. Notably, a common inflection point was observed on the performance curve at flow coefficients of about 0.10 and 0.21. The results of the numerical analyses of the reference and simplified models confirmed that both models provided almost identical predictions, from the design flow point to the critical flow point. However, we observed significant differences in the low-flow region. Particularly, we observed that these differences became evident at flow coefficients of < 0.2 on the performance curve, indicating that the de-swirler significantly impacted the performance in the low-flow region. We confirmed that the surge phenomenon in the low-flow region of the performance curve caused an increase in the incidence angle of the fluid, resulting in unstable flow phenomena, such as flow separation, and recirculation, which adversely affected the performance of the pump. These phenomena were induced by an increase in the flow angle of incidence. A previous related study^32^ revealed that the performance and stability of a pump can be improved with an anti-stall fin, similar to the de-swirler component considered in this study when such phenomena occur at the inlet section of fluid machinery. Therefore, we can infer that the de-swirler also contributed to the partial improvement of unstable flow phenomena in the low-flow region.

Our analysis of the performance characteristic of the variable IGV was based on the simplified model (Fig. 3b). Therefore, the results offered slightly lower predictions in the low-flow region, such as the surging region, of the performance curve. However, it can be seen that in the low flow rate area of the performance curve, the axial pump shows a positive slope that causes unstable operation due to periodic changes in pressure and flow rate. In addition, it is judged that the de-swirler removal and the simplification of the flow path cause differences in performance prediction in the low flow rate area. The flow coefficient of about 0.21 points or more, including design points, tends to be generally consistent with the reference model, and the reliability of the simplified model has already been proven and reported in related prior studies^33,34^.

## Result

### Performance curve evaluation

**Figure 8:**
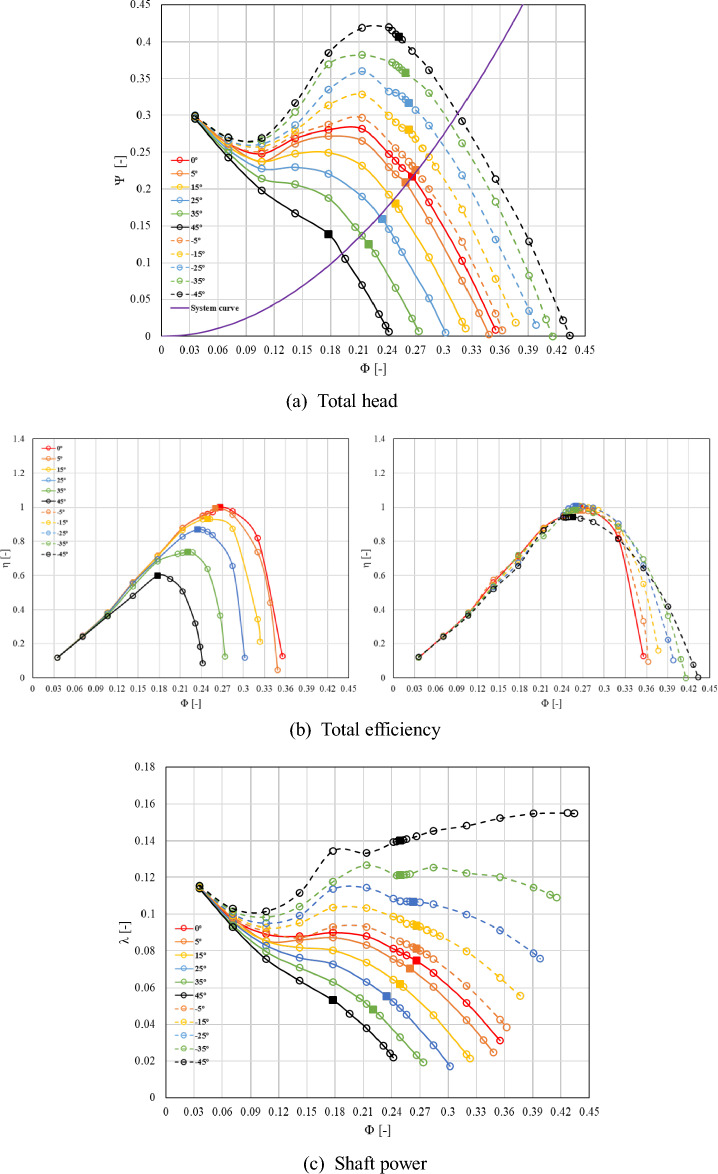
Performance curve at each inlet flow angle.

Figure 8 shows a comparison of the performance curves of the selected simplified model, illustrating the performance variations due to changes in the absolute flow angle at the inlet, which is the focus of this study. Figure 8a represents the performance curves of total head, (b) efficiency, and (c) shaft power. The operating points corresponding to the best efficiency point on the performance curve for each inlet flow angle are indicated by square symbols. The efficiency graph (Fig. 8b) shows the efficiency curves corresponding to the flow angles in the rotational direction ( +) and reverse rotational direction ( −) with respect to the reference flow angle, 0°. The efficiency curve was normalized and expressed based on the best efficiency point of the pump inlet flow angle of 0°. The theoretical system curve was predicted using the quadratic equation using the total head and the flow rate at the best efficiency point of the inlet flow angle 0°.

From the performance curve of the total head, it was observed that as the flow angle gradually increased from the reference flow angle of 0°, the performance decreased across the entire range of flow rates, including the design flow point. Conversely, as the flow angle decreased, the performance increased gradually. It is known that the positive gradient caused by the increase in the incident angle arises from a flow coefficient of about 0.24 when the inlet flow angle is − 45° based on the design flow point. This phenomenon occurs at lower flow points as the inlet flow angle increased gradually; it was not observed when the flow angle exceeded 25°. Therefore, the operation of pumps in the low-flow range results in the periodic fluctuations of flow rate and pressure, leading to the occurrence of an unstable phenomenon. This phenomenon generates noise and vibrations within the pump, impacting its lifespan, and performance. As the inlet flow increased along the rotation direction ( +), the positive gradient variations in the curve of head coefficient decreased. Particularly, the positive gradient variations in the total head curve disappeared when the inlet flow angle exceeded 25°. The operational flow range for actual pump operation, excluding the occurrence region of the surge phenomenon, increased generally in the high-flow region as the flow angle decreased from 0° with respect to the inlet flow angle. Conversely, as the flow angle increased, it gradually decreased into the low-flow region.

The efficiency curve revealed a significant movement of the best efficiency point as the inlet flow angle increases in the rotational direction. As the inlet flow angle increased with respect to 0°, the flow coefficient point corresponding to the best efficiency point decreased. Similarly, the absolute value of the efficiency decreased. As the angle decreased relative to the inlet flow angle of 0°, the variations in the efficiency curve, as well as the shift of the best efficiency point, became relatively less evident. Based on the analysis, when the inlet flow angle changed along the opposite direction ( −) of rotation, the operating region increased as the high flow rate region, although it barely impacted the efficiency. As the flow angle increased from 0° to 45°, the normalized flow coefficient at the best efficiency point decreased by about 0.1, and the normalized efficiency decreased by about 0.4. The efficiency was normalized with respect to the best efficiency point at an inlet flow angle of 0°. The performance curve for shaft power exhibited a similar trend to the performance curve for total head. Our findings revealed that unnecessary power consumption can be reduced by avoiding overload operation when adjusting the flow angle based on the change in the inlet flow rates from the curve of shaft power. Furthermore, efficient pump operation scenarios can be formulated based on the data of the curve of shaft power by installing IGV in the pump and operating it. As the inlet flow angle increased, the operating flow rate, total head, and shaft power at the best efficiency point decreased, and as the inlet flow angle decreased, the change at the best efficiency point became insufficient even though the operating flow region expanded. In the future, when a variable IGV is installed in an axial pump, shaft power can be reduced at the same flow rate by changing the flow angle. By utilizing the variable IGV, the region on the performance curve where the positive curve emerges in the low flow rate range can be avoided. Additionally, in case of an increasing inflow rate, it allows for an expanded operating range through angle adjustments. The optimal angle that must be adjusted using IGV can be predicted based on the inflow rate from a virtual-system curve, thereby avoiding overload operation relative to the inflow rate. Furthermore, establishing operational scenarios that respond to demand fluctuations, based on which the pump can be stably operated and unnecessary shaft power consumption can be reduced, can ensure effective pump operation.

### Internal flow analysis of the axial pump

**Figure 9:**
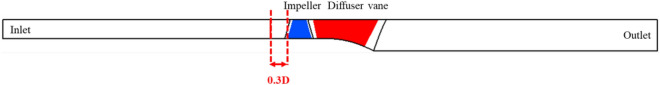
Meridional views of the specific planes of the velocity components.

**Figure 10:**
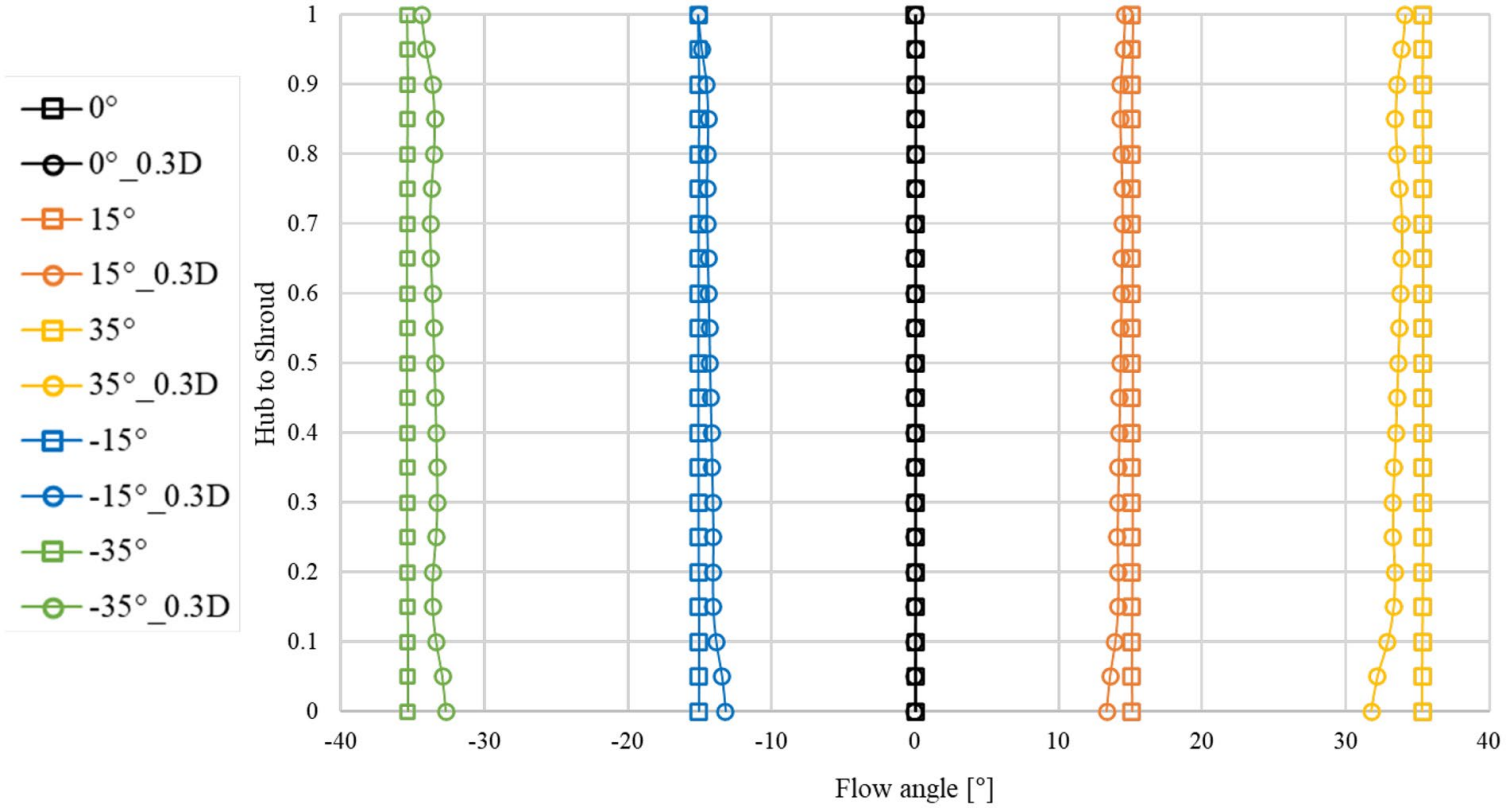
Distribution of the flow angles at the inlet and 0.3D plane.

Figure 9 shows the positions of velocity component distribution analysis according to the variation of the flow angle at the axial-flow pump inlet. The velocity components were analyzed at a position, 0.3D, from the leading edge of the inlet and impeller hub regions of the pump. Figure 10 show the flow angle, axial velocity component, and tangential velocity component at the inlet region and 0.3D from the impeller at the best efficiency points of flow angles 0°, 15°, − 15°, 35°, and − 35°. On the curve, the 0–1 range on the y-axis represents the numerical analysis domain from the hub region to the shroud region. We normalized the velocity components using the ideal values calculated based on the inlet area of the pump and mass flow rate at the best efficiency point for each inlet flow angle, along with the numerical analysis results. The tangential velocity component was normalized using the ideal and absolute values of the tangential theta at each inlet flow angle compared with the numerical analysis values.

**Figure 11:**
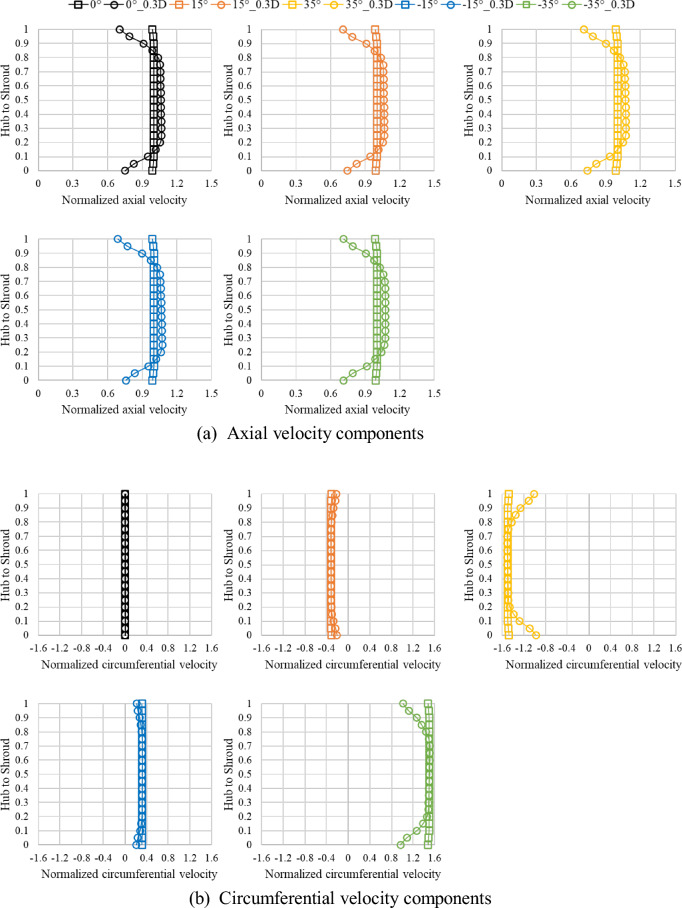
Distributions of the velocity components at the inlet and 0.3D plane.

In Fig. 10, when the inlet flow angle is 0°, the flow angle at the inlet and 0.3D position is the angles same. However, when the inlet flow angle changes, it may be seen that the flow angle at the inlet and the 0.3D position were angles different. As the inlet flow angle gradually changes, the angles at the inlet section and 0.3D position exhibit differences due to distortion phenomena caused by the flow progression from the inlet. However, it can be observed that this difference is relatively small, with a maximum of approximately 2.5°. From Fig. 11a, it can be observed that regardless of the variation in the inlet flow angle, the trend of the axial velocity component remains similar. Furthermore, at the 0.3D position, it was evident that the hub and shroud regions are influenced by wall shear stress, as indicated by the axial velocity component. From Fig. 11b, it can be observed that as the inlet flow angle varies from 0° to 35° and − 35°, the influence of the wall at the 0.3D position becomes more pronounced. Additionally, it was found that the velocity component exhibits a similar trend for both positive and negative angle variations. During numerical analysis, the inlet region was extended for convergence, which is believed to cause a difference from the angle given to the inlet due to the influence of the wall while the flow proceeds from the inlet to the impeller region.

**Figure 12:**
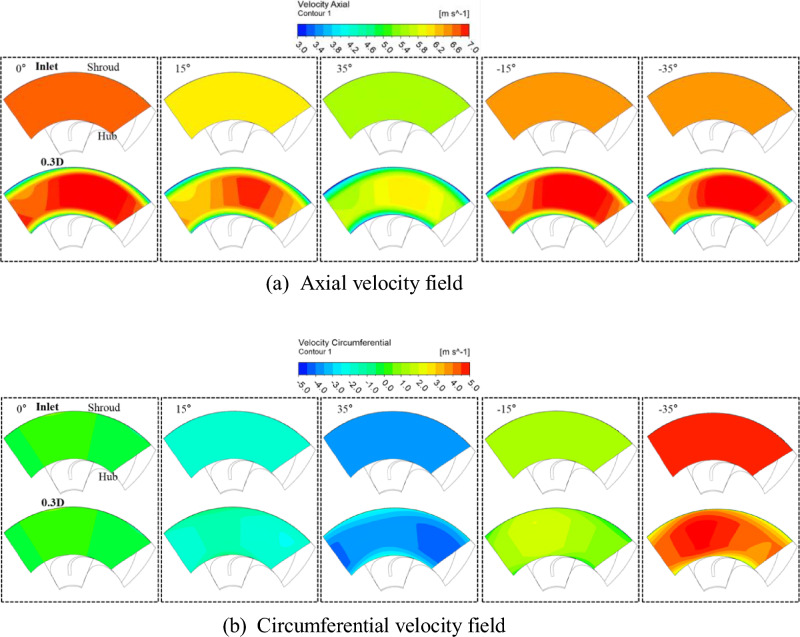
Distributions of the velocity field at the axial pump inlet and 0.3D plane.

Figure 12 shows the inlet at the best efficiency point and the axial and circumferential velocity fields at the 0.3D position when the axial pump inlet flow angles are 0°, 15°, − 15°, 35°, and − 35°. The axial velocity field of Fig. 12 shows that the velocity component in the inlet region is uniform, as shown in Fig. 11a, and the velocity decreases in the hub and shroud region due to the influence of wall shear stress at the 0.3D position. Since the inlet mass flow rate for each inlet flow angle is different, the distribution of the axial velocity field is not the same. It can be seen that the tendency of the inlet and 0.3D position flow distribution is similar as shown in Fig. 11, regardless of the positive and negative angle changes in the angle change of the same size. Through the tangential velocity field, similar to Fig. 11b, it is observed that as the variation in inlet flow angle increases, the velocity difference is non-uniform distributed in the hub and shroud regions. Although there are differences in the magnitude of velocity components for rotational and revise rotational angle variations, the velocity distribution remains similar for the same angle. As mentioned earlier, despite given absolute flow angle at the inlet, it was observed that the flow angle near the impeller inlet region deviates from the inlet flow angle as the variation in inlet flow angle increases. Furthermore, through the axial and tangential velocity component curve, it was confirmed that as the flow approaches the impeller inlet region, the influence of the wall becomes more significant. However, the difference between the flow angles imposed at the inlet and the flow angles near the impeller region is relatively small, with a maximum deviation of approximately 2.5°. On average, the flow angles remain similar. Depending on the angle change, the flow rate at the best efficiency point varies, so there is a difference in the size of the internal flow field velocity component, but the flow distribution is similar. Through this, it is predicted that there will be a difference in the actual case of installation of the inlet guide vane (IGV) and the results of numerical analysis, but it is judged that there will be possible in predicting the effect of the variable IGV. Therefore, it is judged that the results of this study can be used to predict the performance before installing the variable IGV and establish a pump operation scenario.

**Figure 13:**
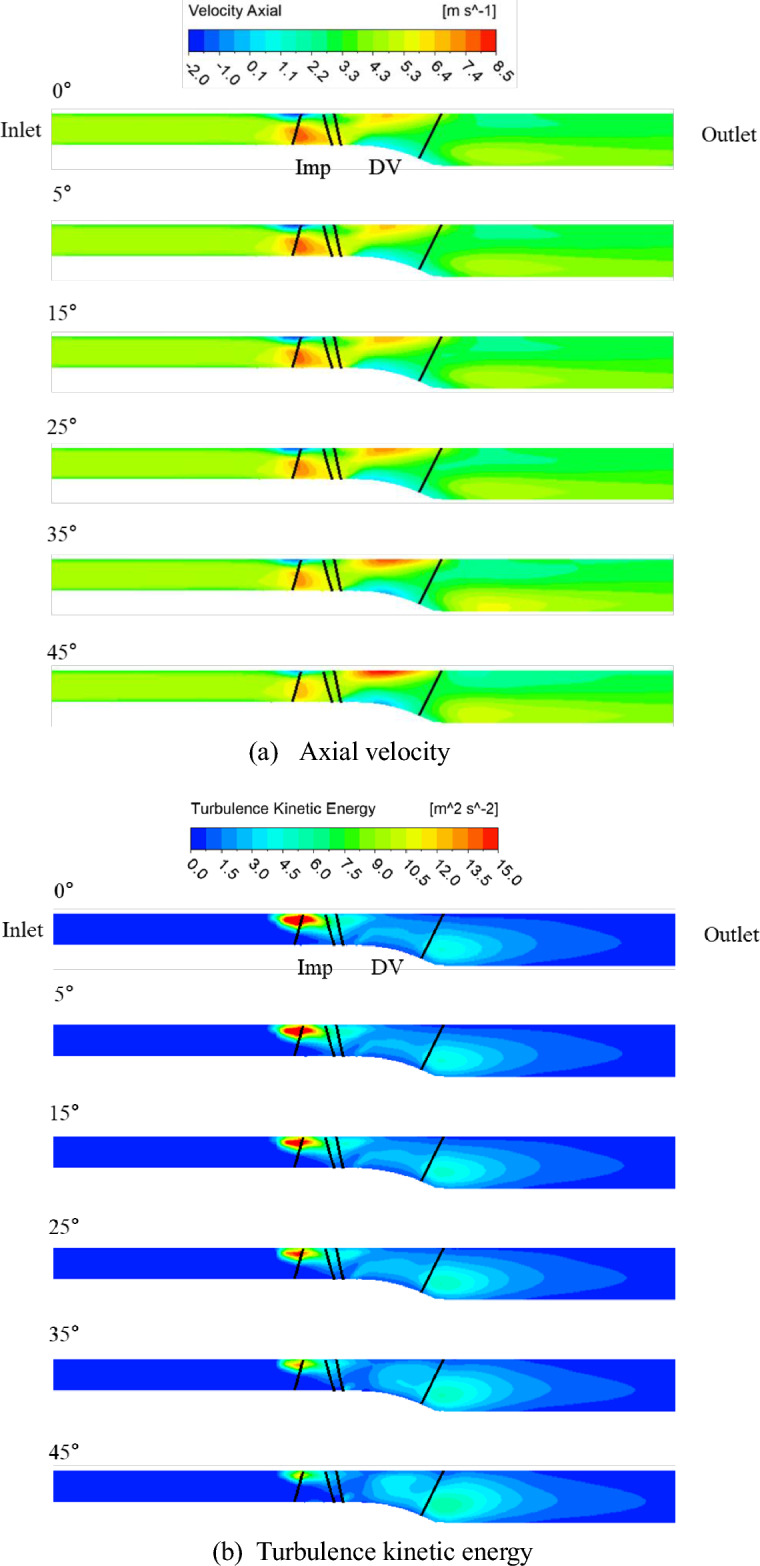
Distribution of the flow field at a flow coefficient of 0.17.

**Figure 14:**
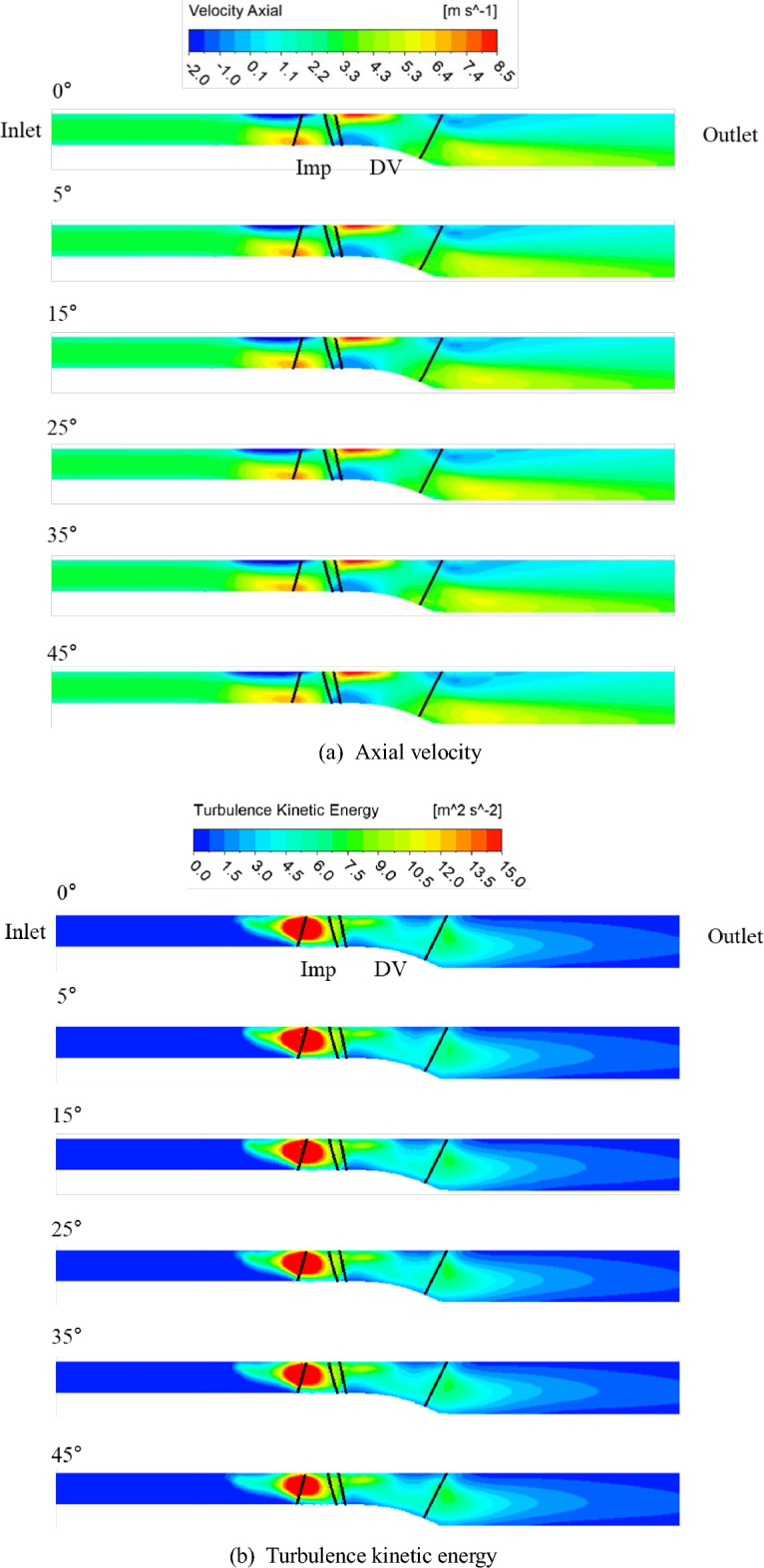
Distribution of the flow field at a flow coefficient of 0.1.

Figures 13 and 14 show the axial velocity and turbulence kinetic energy fields along the meridional plane corresponding to the changes in the inlet flow angle, respectively. To analyze the internal flow distribution in cases where the positive slope of the curvature disappeared on the meridional curve, only the angles in the rotational direction ( +) was considered. The measurement locations were observed at the flow coefficient of 0.17, where the positive curvature-slope variation decreased with the changes in the inlet flow angle, and at the flow coefficient of 0.1, which corresponds to the point with the deepest positive curvature variation. Based on Fig. 8a, which shows the performance curve of *H*, we observed that the positive curvatures of 0.17 and 0.1 were mitigated as the inlet flow angle varied from 0° to 45°. The flow separation phenomenon, observed in the shroud region at an inlet flow angle of 0°, as shown in Fig. 13. The elimination of flow separation results in smoother flow in the shroud region, leading to a decrease in the previously dominant axial velocity component at the impeller inlet hub and mid span region. It can be seen that the turbulence kinetic energy, which may be observed in the shroud region of the flow angle of 0°, gradually decreases as it changes to 45°. Therefore, at the flow coefficient of 0.17, it can be seen that the positive slope and unstable flow phenomenon that occur when the inlet flow angle is 0° change to 45°, and the positive slope relaxation and internal flow change stably. Therefore, it was confirmed that the pump can be operated stably by changing the flow angle in the operation area that was not stable when the existing flow angle was 0°.

Figure 14 shows that the flow coefficient 0.1 point, which is the deepest point of the positive slope from the performance curve, gradually changes the positive slope as the inlet flow angle changes from 0 to 45°, but the internal flow field is still unstable. It can be seen that there is no significant change in the flow separation phenomenon and turbulence kinetic energy generated in the shroud region as the inlet flow angel changes to 45°. It is judged that it is necessary to analyze it in more detail by performing unsteady state analysis.

## Conculsions

The main objective of this study is to predict the performance, as well as analyze the surge phenomenon before actually installing IGV at the inlet of an axial-flow pump. Numerical analysis was performed to simulate the swirling flow occurring downstream of IGV, and an absolute flow angle was assigned at the pump inlet. Thereafter, performance evaluation, and analysis of the internal flow were performed based on these simulations.

First, as the flow angle at the axial pump inlet changed in the opposite direction ( −) of rotation, the operating flow range increased. However, dissimilar to the total head curve, the efficiency curve exhibited a smaller magnitude variation. As the inlet flow angle changed into an angle in the rotational direction ( +), the positive slope change caused by the surge phenomenon in the low-flow region decreased, and no positive slope change was observed in the low-flow region when the inlet flow angle was 25°.

Second, as the inlet flow angle changed from 0° to 45° in the positive direction, the flow separation and turbulence kinetic energy in the region decreased significantly at a flow coefficient of about 0.17. This improved the positive slope and unstable internal flow in the low-flow region with an inlet flow angle of 0° by changing the flow angle, indicating that the existing unstable operating region changed stably by changing the flow angle.

Third, when an absolute flow angle was assigned at the inlet, the inlet flow angle near the impeller inlet differed from the one at the inlet as the variation in the flow angle increased. The axial and tangential velocity component curves revealed that as the flow progressed toward the impeller inlet, it was influenced by the wall shear stress in the hub and shroud regions. However, the distributions of the internal flow field according to the average angle and angle changes were similar. Therefore, it can be used to predict the performance of IGV.

Fourth, It can be seen that the positive slope changes smoothly as the inlet angle of the pump changes from 0° to 45° at 0.1 point of the flow coefficient, which is the point where the positive slop is the deepest in the total head performance curve. However, it can be confirmed that there is a back-flow phenomenon and turbulent kinetic energy distribution through the internal flow filed distribution. Therefore, it is necessary to conduct a detailed analysis of the internal flow phenomenon by conducting a transient analysis at 0.1 point, where the positive slope is the deeprst point in the performance curve.

Fifth, based on the analysis of the performance characteristics and internal flow field of the pump, the performance of IGV can be predicted before installing the variable-geometry structure inside an axial-flow pump. In addition, due to the simple flow path shape, it is thought that the results of predicting the performance of IGV can be applied to various sites by giving an absolute flow angle to the pump inlet before the actual IGV installation and can be used to predict operational scenarios. Through this, it is possible to identify the surging phenomenon that occur inside the pump in the low flow rate area during actual pump operation. It is possible to establish an expected operation scenario by predicting the optimal angle according to the fluctuation of the inflow flow rate. Through this, it is believed that an efficient operation scenario for energy reduction can be established, and loss caused during the design and production peocess can be prevented.

## Data Availability

The data that support the findings of this study are available from the corresponding author upon request.
